# Supplementary material for: Macrosymbionts of starfish Echinaster luzonicus (Gray, 1840) in the waters of a volcanic western Pacific island
Source: PLoS One. 2022 Nov 30;17(11):e0278288. doi: 10.1371/journal.pone.0278288 (PMC9710757; doi:10.1371/journal.pone.0278288)
Supplement: Attachment — Submitted filename: MS TI starfish symbionts Response to Reviewers 0829.docx [file pone.0278288.s002.docx]

**Author responses to referee comments (29^th^ August 2022)**

**Date:** Jul 18 2022 03:12PM

**To:** "Jiang-Shiou Hwang" [jshwang@mail.ntou.edu.tw;jshwang@ntou.edu.tw](mailto:jshwang@mail.ntou.edu.tw;jshwang@ntou.edu.tw)

**From:** "PLOS ONE" [plosone@plos.org](mailto:plosone@plos.org)

**Subject:** PLOS ONE Decision: Revision required [PONE-D-21-26592]

PONE-D-21-26592

Macrosymbionts of starfish *Echinaster luzonicus* (Gray, 1840), with a new biogeographic record of scaleworm *Asterophilia carlae* Hanley, 1989, in the waters of a volcanic western Pacific island

PLOS ONE

Dear Dr. Hwang,

Thank you for submitting your manuscript to PLOS ONE. After careful consideration, we feel that it has merit but does not fully meet PLOS ONE’s publication criteria as it currently stands. Therefore, we invite you to submit a revised version of the manuscript that addresses the points raised during the review process.

1. Data in the current manuscript are from the year 2020. More data are needed for this work.

2. The "Discussion" part needs in-depth-discussion.

3. This manuscript needs a suitable title.

4. All figures should be added the scale bar.

If you plan to submit a revision, the revision will be sent to previous reviewers for further consideration.

Please submit your revised manuscript by Sep 01 2022 11:59PM. If you will need more time than this to complete your revisions, please reply to this message or contact the journal office at [plosone@plos.org](mailto:plosone@plos.org). When you're ready to submit your revision, log on to <https://www.editorialmanager.com/pone/> and select the 'Submissions Needing Revision' folder to locate your manuscript file.

Please include the following items when submitting your revised manuscript:

- A rebuttal letter that responds to each point raised by the academic editor and reviewer(s). You should upload this letter as a separate file labeled 'Response to Reviewers'.
- A marked-up copy of your manuscript that highlights changes made to the original version. You should upload this as a separate file labeled 'Revised Manuscript with Track Changes'.
- An unmarked version of your revised paper without tracked changes. You should upload this as a separate file labeled 'Manuscript'.

If you would like to make changes to your financial disclosure, please include your updated statement in your cover letter. Guidelines for resubmitting your figure files are available below the reviewer comments at the end of this letter.

If applicable, we recommend that you deposit your laboratory protocols in protocols.io to enhance the reproducibility of your results. Protocols.io assigns your protocol its own identifier (DOI) so that it can be cited independently in the future. For instructions see: <https://journals.plos.org/plosone/s/submission-guidelines#loc-laboratory-protocols>. Additionally, PLOS ONE offers an option for publishing peer-reviewed Lab Protocol articles, which describe protocols hosted on protocols.io. Read more information on sharing protocols at <https://plos.org/protocols?utm_medium=editorial-email&utm_source=authorletters&utm_campaign=protocols>.

We look forward to receiving your revised manuscript.

Kind regards,

Wan-Xi Yang, Ph.D.
Academic Editor
PLOS ONE

**Authors’ response:** We are thankful for the very constructive comments and efforts from two reviewers. We fully agree with the suggestions and have corrected them accordingly and hopefully eradicated errors.

Journal Requirements:

When submitting your revision, we need you to address these additional requirements.

1. Please ensure that your manuscript meets PLOS ONE's style requirements, including those for file naming. The PLOS ONE style templates can be found at 
<https://journals.plos.org/plosone/s/file?id=wjVg/PLOSOne_formatting_sample_main_body.pdf> and 
<https://journals.plos.org/plosone/s/file?id=ba62/PLOSOne_formatting_sample_title_authors_affiliations.pdf>

2. In your Methods section, please provide additional information regarding the permits you obtained for the work. Please ensure you have included the full name of the authority that approved the field site access and, if no permits were required, a brief statement explaining why.

3. Thank you for stating the following financial disclosure: "Financial support from the Ministry of Science and Technology (MOST) of Taiwan through grant no. MOST 108-2811-M-019-504, MOST 109-2811-M-019-504 and MOST 110-2811-M-019-504 to L.-C. Tseng, as well as grant no. MOST 106-2621-M-019-001, MOST 107-2621-M-019-001, MOST 108-2621-M-019-003, MOST 109-2621-M-019-002, and MOST 110-2621-M-019-001, and Center of Excellence for Ocean Engineering (Grant No. 109J13801-51) to J.-S. Hwang. The funders had no role in study design, data collection and analysis, decision to publish, or preparation of the manuscript."

We note that one or more of the authors is affiliated with the funding organization, indicating the funder may have had some role in the design, data collection, analysis or preparation of your manuscript for publication; in other words, the funder played an indirect role through the participation of the co-authors. If the funding organization did not play a role in the study design, data collection and analysis, decision to publish, or preparation of the manuscript and only provided financial support in the form of authors' salaries and/or research materials, please do the following:

a. Review your statements relating to the author contributions, and ensure you have specifically and accurately indicated the role(s) that these authors had in your study. These amendments should be made in the online form.

b. Confirm in your cover letter that you agree with the following statement, and we will change the online submission form on your behalf:

“The funder provided support in the form of salaries for authors [insert relevant initials], but did not have any additional role in the study design, data collection and analysis, decision to publish, or preparation of the manuscript. The specific roles of these authors are articulated in the ‘author contributions’ section.

4. In your Data Availability statement, you have not specified where the minimal data set underlying the results described in your manuscript can be found. PLOS defines a study's minimal data set as the underlying data used to reach the conclusions drawn in the manuscript and any additional data required to replicate the reported study findings in their entirety. All PLOS journals require that the minimal data set be made fully available. For more information about our data policy, please see <http://journals.plos.org/plosone/s/data-availability>.

Upon re-submitting your revised manuscript, please upload your study’s minimal underlying data set as either Supporting Information files or to a stable, public repository and include the relevant URLs, DOIs, or accession numbers within your revised cover letter. For a list of acceptable repositories, please see <http://journals.plos.org/plosone/s/data-availability#loc-recommended-repositories>. Any potentially identifying patient information must be fully anonymized.

Important: If there are ethical or legal restrictions to sharing your data publicly, please explain these restrictions in detail. Please see our guidelines for more information on what we consider unacceptable restrictions to publicly sharing data: <http://journals.plos.org/plosone/s/data-availability#loc-unacceptable-data-access-restrictions>. Note that it is not acceptable for the authors to be the sole named individuals responsible for ensuring data access.

We will update your Data Availability statement to reflect the information you provide in your cover letter.

5. Please upload a copy of Supporting Information S1 file which you refer to in your text on page 24.

[Note: HTML markup is below. Please do not edit.]

**Review Comments to the Author**

**Reviewer #1:**

After reading the whole manuscript, I do not see the reasons to why scaleworm *Asterophilia carlae* should appear in the title as there are other new findings as well. What is so special with scaleworm *Asterophilia carlae*?

**Authors’ response:** We are thankful for the constructive comments. Since scaleworm *Asterophilia carlae* was found for the first time in the study waters, therefore its name was specifically put in the title. We changed the title to “Macrosymbionts of starfish *Echinaster luzonicus* (Gray, 1840) in the waters of a volcanic western Pacific island”.

Results were well presented and discussed. However, I suggest discussion to start with general observation followed by specific data. Therefore, from lines 277 to 290 where the others are discussing general observation, should have stated earlier.

**Authors’ response:** We are thankful for the very constructive comments and efforts from the reviewer. In the corrected version, we have rearranged the paragraphs and descriptions as suggested.

Line 155: I don’t know the reason why the others present issues of medicinal value of the species Echinaster luzonicus. I think the others were supposed to confine themselves in distribution and variability among the study species especially in this paragraph.

**Authors’ response:** Thanks for the useful advice. This sentence has been deleted as suggested.

Compare lines 157-159 and lines 130-132: While previous studies show different macrosymbionts to coexist on *E. luzonicus*, the findings of this study shows each individuat starfish to host one species of macrosymbiont. I therefore expected this to be discussed here.

**Authors’ response:** The term "coexist" means that these symbiotic animals have all been recorded on starfish *E. luzonicus*. These references do not record the presence of two coexisting animals on a single starfish individual.

**Reviewer #2:**

The data of the ms are only from 2020, so the results are somewhat accidental and simple, the reliability need to be considered. It is recommended to increase the sampling time. All figures should be added the scale bar.

**Authors’ response:** We are thankful for the suggestions. Due to research funding constraints, we can only conduct the survey in 2020, so we do not have survey data for 2021 to the present. The survey will continue in the future with the support of funding. In July of this year (2022), we visited the surveyed reefs again and only recorded *Zenopontonia soror* symbiosis with starfish *E. luzonicus*. So far, there are very few records of *Asterophilia carlae*, and probably its population is not abundant, so the chance of discovery is also very low. In 2020, both surveys covered more than 90% of the reef area, and the results obtained can definitely be relied on. We have added a scale bar in Figures 3-5 in the corrected version of the manuscript.
